# Single-Dose Liposomal Amphotericin B Treatment for Cryptococcal Meningitis

**DOI:** 10.1056/NEJMoa2111904

**Published:** 2022-03-24

**Authors:** Joseph N Jarvis, David S Lawrence, David B Meya, Enock Kagimu, John Kasibante, Edward Mpoza, Morris K Rutakingirwa, Kenneth Ssebambulidde, Lillian Tugume, Joshua Rhein, David R Boulware, Henry C Mwandumba, Melanie Moyo, Henry Mzinganjira, Cecilia Kanyama, Mina C Hosseinipour, Chimwemwe Chawinga, Graeme Meintjes, Charlotte Schutz, Kyla Comins, Achita Singh, Conrad Muzoora, Samuel Jjunju, Edwin Nuwagira, Mosepele Mosepele, Tshepo Leeme, Keatlaretse Siamisang, Chiratidzo E Ndhlovu, Admire Hlupeni, Constantine Mutata, Erik van Widenfelt, Tao Chen, Duolao Wang, William Hope, Timothée Boyer-Chammard, Angela Loyse, Síle F Molloy, Nabila Youssouf, Olivier Lortholary, David G Lalloo, Shabbar Jaffar, Thomas S Harrison

**Affiliations:** 1 Department of Clinical Research, Faculty of Infectious and Tropical Diseases, London School of Hygiene and Tropical Medicine, London, UK; 2 Botswana Harvard AIDS Institute Partnership, Gaborone, Botswana; 3 Infectious Diseases Institute, College of Health Sciences, Makerere University, Kampala, Uganda; 4 University of Minnesota, Minneapolis, Minnesota, USA; 5 Department of Medicine, School of Medicine, Makerere University, Kampala, Uganda; 6 Liverpool School of Tropical Medicine, Liverpool, UK; 7 Malawi-Liverpool-Wellcome Clinical Research Programme, Blantyre, Malawi; 8 Department of Medicine, Kamuzu University of Health Sciences, Blantyre, Malawi; 9 Lilongwe Medical Relief Trust (UNC Project), Lilongwe, Malawi; 10 Department of Medicine, University of North Carolina, Chapel Hill, North Carolina, USA; 11 Wellcome Centre for Infectious Diseases Research in Africa (CIDRI-Africa), Institute of Infectious Disease and Molecular Medicine, University of Cape Town, Cape Town, South Africa; 12 Department of Medicine, University of Cape Town, Cape Town, South Africa; 13 Department of Radiology, Groote Schuur Hospital, Cape Town, South Africa; 14 Mbarara University of Science and Technology, Mbarara, Uganda; 15 Department of Internal Medicine, University of Botswana, Gaborone, Botswana; 16 Department of Family Medicine and Public Health, University of Botswana, Gaborone, Botswana; 17 Department of Health Services Management, Ministry of Health and Wellness, Gaborone, Botswana; 18 Internal Medicine Unit, Faculty of Medicine and Health Sciences, University of Zimbabwe, Harare, Zimbabwe; 19 Department of Public Health, Policy & Systems, Institute of Population Health, University of Liverpool, Liverpool, UK; 20 Antimicrobial Pharmacodynamics and Therapeutics, University of Liverpool, Liverpool, UK; 21 Institut Pasteur, CNRS, Molecular Mycology Unit and National Reference Center for Invasive Mycoses and Antifungals, UMR 2000, Paris, France; 22 Université de Paris, Necker Pasteur Center for Infectious and Tropical Medicine, Hôpital Necker Enfants Malades, AP-HP, IHU Imagine, Paris, France; 23 Institute of Infection and Immunity, St George’s University London, London, UK; 24 Clinical Academic Group in Infection and Immunity, St George’s University Hospitals NHS Foundation Trust, London, UK; 25 MRC Centre for Medical Mycology, University of Exeter, Exeter, UK

## Abstract

**Background:**

Cryptococcal meningitis is a leading cause of HIV-related mortality in sub-Saharan Africa. Based on phase-II data, we performed a phase-III randomized controlled non-inferiority trial to determine the efficacy of a single high-dose liposomal amphotericin B based treatment regimen.

**Methods:**

HIV-positive adults with cryptococcal meningitis in Botswana, Malawi, South Africa, Uganda and Zimbabwe were randomized 1:1 to induction therapy of either (i) single, high-dose liposomal amphotericin B 10mg/kg given with 14 days of flucytosine 100mg/kg/day and fluconazole 1200mg/day (AmBisome group), or (ii) the current WHO recommended treatment of 7 daily doses of amphotericin B deoxycholate (1mg/kg/day) plus flucytosine (100mg/kg/day), followed by 7 days of fluconazole 1200mg/day (control group). The primary endpoint was all-cause mortality at 10 weeks with the trial powered to show non-inferiority at a 10% margin.

**Results:**

We randomized 844 participants. None were lost-to-follow-up. In intention-to-treat analysis, 10-week mortality was 24.8% (101 of 407; 95% confidence interval [CI] 20.7-29.3%) in the AmBisome group and 28.7% (117 of 407; 95% CI 24.4-33.4%) in controls. The absolute difference in mortality was -3.9%, with an upper 1-sided 95% confidence interval of 1.2%. Fungal clearance from cerebrospinal fluid was -0.40 log_10_ CFU/ml/day in the AmBisome group and -0.42 log_10_ CFU/ml/day in the control group. Fewer participants experienced grade 3 or 4 adverse events in the AmBisome group than the control group (50.0% vs. 62.3%).

**Conclusions:**

Single dose liposomal amphotericin B (10mg/kg) on a backbone of flucytosine and fluconazole was non-inferior to the current WHO recommended standard of care for HIV-associated cryptococcal meningitis and associated with fewer adverse events. **(Trial registration number:** ISRCTN72509687.)

Cryptococcal meningitis is the most frequent cause of adult meningitis in areas with high human immunodeficiency virus (HIV) prevalence^1-3^ and is the second leading cause of HIV-related mortality worldwide, with the majority of deaths occurring in sub-Saharan Africa.^4^ Despite widened access to antiretroviral therapy (ART), there is a persistent burden of advanced HIV disease in the region,^5-7^ and the number of cryptococcal meningitis cases remains high.^7,8^

Poor outcomes with conventional antifungal treatment regimens are a key driver of the high cryptococcal meningitis mortality, with high rates of toxicity seen with commonly used 2-week amphotericin B deoxycholate-based regimens, and poor efficacy with fluconazole monotherapy which is associated with 10-week mortality rates in excess of 50%.^9,10^ In 2018 the World Health Organization (WHO) updated international guidelines to recommend induction therapy with the less toxic and more efficacious one week regimen of amphotericin B deoxycholate and flucytosine in resource limited settings,^11^ following publication of the ACTA trial.^12^ However, even one week of amphotericin B deoxycholate is associated with anemia, kidney impairment, and electrolyte abnormalities,^9^ and administering and monitoring seven days of intravenous amphotericin poses logistical challenges in many clinical settings.

Liposomal amphotericin (L-AmB) is potentially well suited for use in short-course induction treatment for cryptococcal meningitis as it can be given at higher doses owing to lower rates of drug-induced toxicity^13-15^, has a long tissue half-life,^13,14,16-18^ and effectively penetrates into brain tissue.^13,19,20^ The concept of single, high-dose L-AmB has been established in the treatment of visceral leishmaniasis,^21^ and pharmacokinetic data from animal models and humans indicate that increasing L-AmB dosing from the currently recommended 3–4 mg/kg may lead to improved outcomes in cryptococcal meningitis, and that short-course regimens may be as effective as daily therapy.^16,17,22,23^ A phase-II clinical trial examining the efficacy of single high-dose L-AmB, two high-doses of L-AmB given on days 1 and 3, or three high-doses of L-AmB given on days 1, 3, and 7, compared with 14 daily 3 mg/kg doses of L-AmB, all given with 14 days of high dose fluconazole, showed that the rate of fungal clearance from the cerebrospinal fluid (CSF) in all three short-course, high-dose arms was non-inferior to the control arm.^24^ Maximal fungicidal activity was achieved with a single 10mg/kg L-AmB dose with no evidence of additional benefit with further doses, in keeping with animal model data.^23,25^ No safety concerns have been identified with high-dose L-AmB compared with prior experience in trials using amphotericin B deoxycholate.^9,24^

Based on the findings of the phase-II trial^24^ and phase-III trial data showing the role of flucytosine in induction treatment of cryptococcal meningitis,^12^ we conducted an open label phase-III randomized controlled non-inferiority trial testing a single 10mg/kg high-dose of L-AmB given with oral flucytosine and fluconazole for two weeks^12^ against the recommended WHO first-line induction treatment of one-week amphotericin B deoxycholate plus flucytosine followed by one week of high-dose fluconazole.

## Methods

### Trial design and participants

The study design has been described previously^26^ and is provided in the full study protocol available as Supplementary Material at nejm.org. HIV-positive adults (≧18 years) with a first episode of cryptococcal meningitis, diagnosed by positive India Ink or cryptococcal antigen (CrAg lateral flow assay, IMMY, Norman, Oklahoma, USA) in CSF, were recruited from eight Hospitals: Princess Marina Hospital, Gaborone, Botswana; Queen Elizabeth Central Hospital, Blantyre and Kamuzu Central Hospital, Lilongwe, Malawi; Mitchells Plain Hospital and Khayelitsha Hospital, Cape Town, South Africa; Kiruddu National Referral Hospital, Kampala and Mbarara Regional Referral Hospital, Mbarara, Uganda; and Parirenyatwa Central Hospital, Harare, Zimbabwe. Participants were excluded if they had received more than two doses of either amphotericin or treatment dose fluconazole (≧800mg) prior to screening, declined consent or in cases of impaired capacity to consent had no legal representative to consent on their behalf, were pregnant, breast-feeding, taking contraindicated concomitant drugs, or had any previous adverse reaction to the study drugs. Late exclusion criteria, put in place to enable rapid enrolment of critically unwell participants pending baseline blood test results, were alanine transaminase (ALT) >5 times the upper limit of normal (>200 IU/L), polymorphonuclear leukocytes (PMNs) <500 x 10^6^/L or platelets <50,000 x 10^6^/L.

### Interventions and randomization

Participants were individually randomized to either (i) single dose L-AmB 10mg/kg (AmBisome, Gilead Sciences Inc.) plus 14 days of flucytosine 100mg/kg/day and fluconazole 1200mg/day (the “AmBisome” group, a three-drug regimen^27^), or (ii) amphotericin B deoxycholate (1mg/kg/day) plus flucytosine (100mg/kg/day) for seven days, followed by fluconazole 1200mg/day on days 8-14 (the “control” group, a two-drug regimen), using a computer-generated randomization list with block sizes of four and six, stratified by site, in a 1:1 ratio. Randomization was performed electronically within a bespoke Electronic Data Capture tool in which the allocation sequence was concealed from all study investigators involved in participant recruitment. Treatment group allocation was provided to recruiting teams after consent and enrolment. Allocation was open label. AmBisome was donated by Gilead Sciences Inc; amphotericin B deoxycholate was purchased from Bristol Myers Squibb; flucytosine was purchased from Mylan; and fluconazole was purchased from Cipla/Medopharm. At sites where the Pfizer Diflucan Partnership Program was operational, donated Pfizer fluconazole was used if available. All participants were treated in hospital for a minimum of seven days. L-AmB 10 mg/kg was given in 1 liter of 5% dextrose over 2 hours, and amphotericin B deoxycholate 1 mg/kg in 1 liter of 5% dextrose over 4 hours. Participants received 1L of intravenous normal saline prior to any amphotericin dose, plus further IV fluids of at least one additional liter each day of amphotericin therapy. Potassium and magnesium supplements were given on each day participants received amphotericin and then for two additional days. Oral medications were given by nasogastric tube if participants were unable to swallow. Laboratory blood tests were monitored regularly during the first two weeks and again at week 4 (schedule provided in Supplementary Appendix Table S1). Lumbar punctures were performed at diagnosis, day 7, and day 14 for quantitative cryptococcal cultures. Participants with raised intracranial pressure received additional daily therapeutic lumbar punctures until the pressure was controlled to <20 cm H_2_O. Participants were followed in outpatient clinics for 10 weeks and contacted telephonically at week 16. If participants missed clinic appointments the trial teams traced them either telephonically or in person. After the 2-week induction period all participants received fluconazole 800mg/day for 8 weeks and 200mg/day thereafter. Antiretroviral therapy was initiated, re-initiated or switched at weeks 4-6 and was chosen in accordance with National Guidelines.

### Assessment of outcomes

The primary endpoint was all-cause mortality within 10 weeks after randomization. Secondary end points included: the rate of CSF fungal clearance over the 14 days of induction therapy; the proportion of participants in each arm developing clinical and DAIDS laboratory-defined grade 3/4 adverse events; and median absolute or percentage change from baseline in laboratory defined parameters.

### Ethics and study oversight

The protocol was approved by the London School of Hygiene and Tropical Medicine Research Ethics Committee and by all the site ethics committees and national regulatory bodies. All participants provided written informed consent. In those with abnormal mental status, written consent was obtained from the next-of-kin, and the participants were re-consented on recovery of capacity to consent. An Independent Data Monitoring Committee monitored the study and reviewed the trial data regularly. The trial funder, suppliers and drug manufacturers had no role in the study design, data collection, analysis, interpretation, or manuscript presentation. The authors vouch for the accuracy and completeness of the data and for the adherence of the trial to the trial protocol.

### Statistical analysis

Assuming a 35% 10-week mortality in both arms, a sample size of 390 per arm (780 in total) was calculated to have 90% power to detect an upper limit of the one-sided 95% confidence interval (CI) of the absolute difference in percentages risks to be within 10% (the specified non-inferiority margin). The primary analysis was based on the intention-to-treat population, defined as all randomized study participants who did not meet any late exclusion criteria. A generalized linear model with a binomial distribution was used to calculate mortality differences.

We performed two sensitivity analyses. First, a per-protocol analysis was performed excluding participants who missed more than one day of any single treatment in the first two weeks or more than two weeks of fluconazole consolidation treatment between weeks 2 and 10. Second, we performed adjusted analyses adjusting for pre-specified covariates: site, age, sex, baseline Glasgow Coma Scale, CD4 count, CSF cryptococcal colony forming units/mL, antiretroviral therapy status, hemoglobin and CSF opening pressure. Analysis of log-transformed longitudinal CSF fungal counts was performed using a linear mixed-effects model, in which undetectable measurements were left-censored.^28^ Adverse event analyses were reported for a safety population consisting of all participants who received one or more doses of study medication. Analyses were conducted using SAS® (version 9.4). See Supplementary Appendix for full statistical analysis plan.

## Results

### Trial population

**Figure 1:**
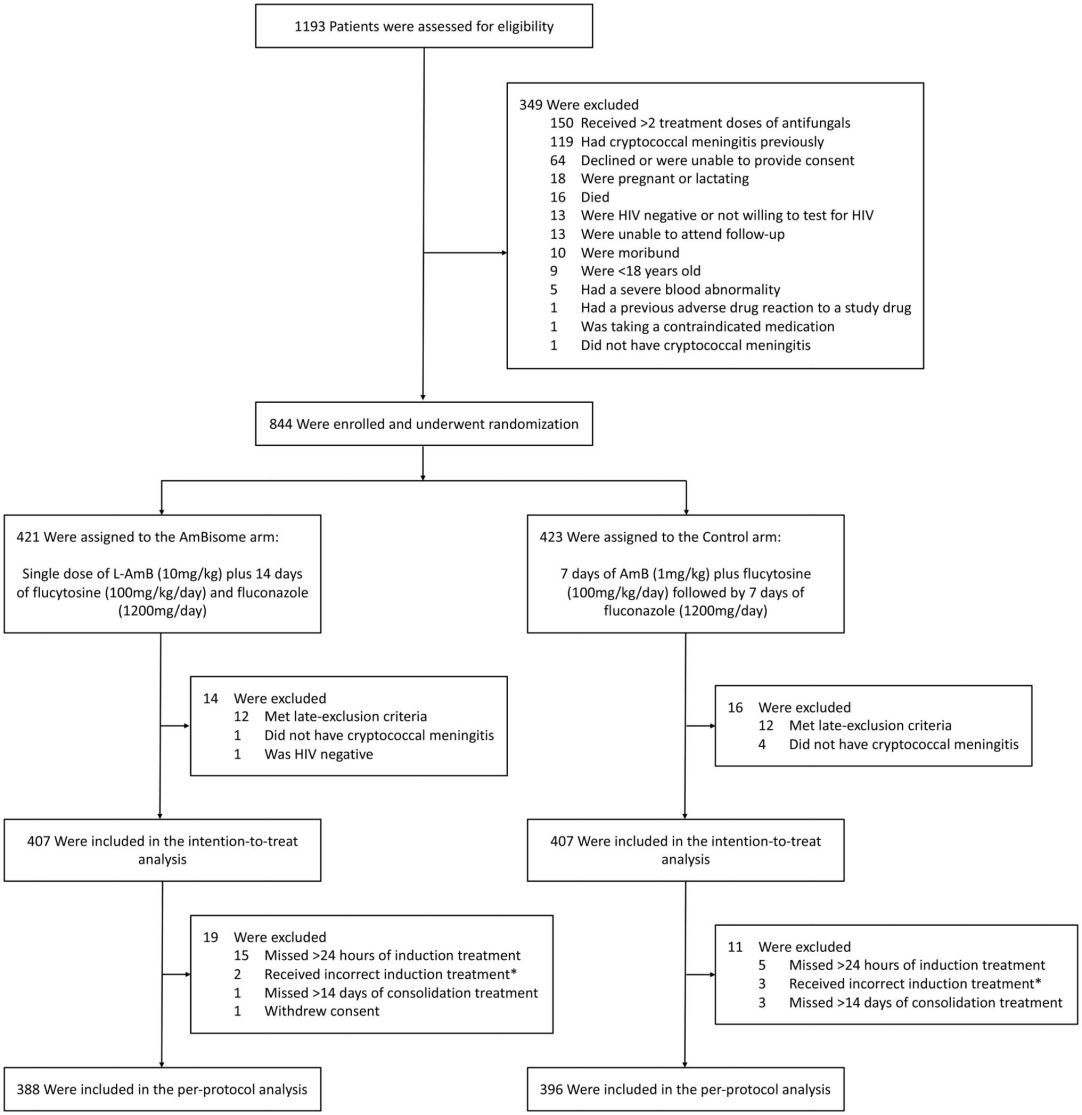
Screening, randomization, and analysis populations (CONSORT). Participants may have had more than one reason for exclusion. AmB denotes amphotericin B deoxycholate, HIV human immunodeficiency virus, and L-AmB liposomal amphotericin. *Two participants in the AmBisome group received at least one dose of amphotericin B deoxycholate and three participants in the control group received high dose fluconazole during the first week of induction therapy.

From January 2018 to February 2021, 844 participants were randomized (Figure 1). Of these, 30 were excluded as: 24 met the pre-defined late exclusion criteria (13 due to low platelets, 4 due to low neutrophils, 2 due to raised ALT, 3 with low platelets and low neutrophils, and 1 with low platelets and raised ALT, Supplementary Appendix Table S2), 5 did not have cryptococcal meningitis, and 1 was HIV negative – leaving 814 participants in the intention-to-treat population. None were lost to follow up. A further 30 participants were excluded from the per-protocol population (20 missed more than one day of treatment in the first two weeks, 6 received incorrect treatment, and 4 missed more than two weeks of fluconazole consolidation treatment between weeks 2 and 10; full listing in Supplementary Appendix Table S2). Baseline characteristics were similar in both arms (Table 1).

### Primary Outcome

**Figure 2:**
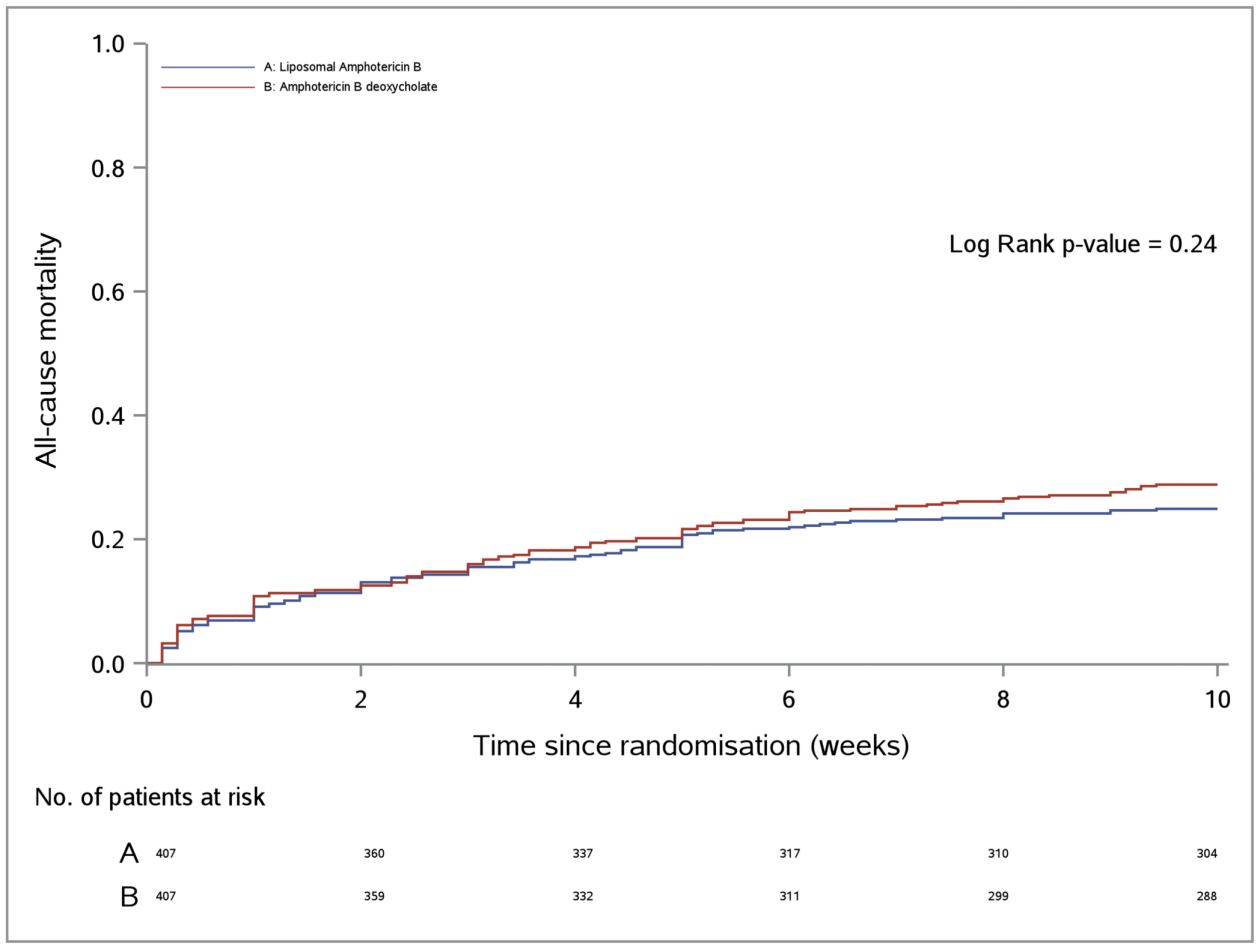

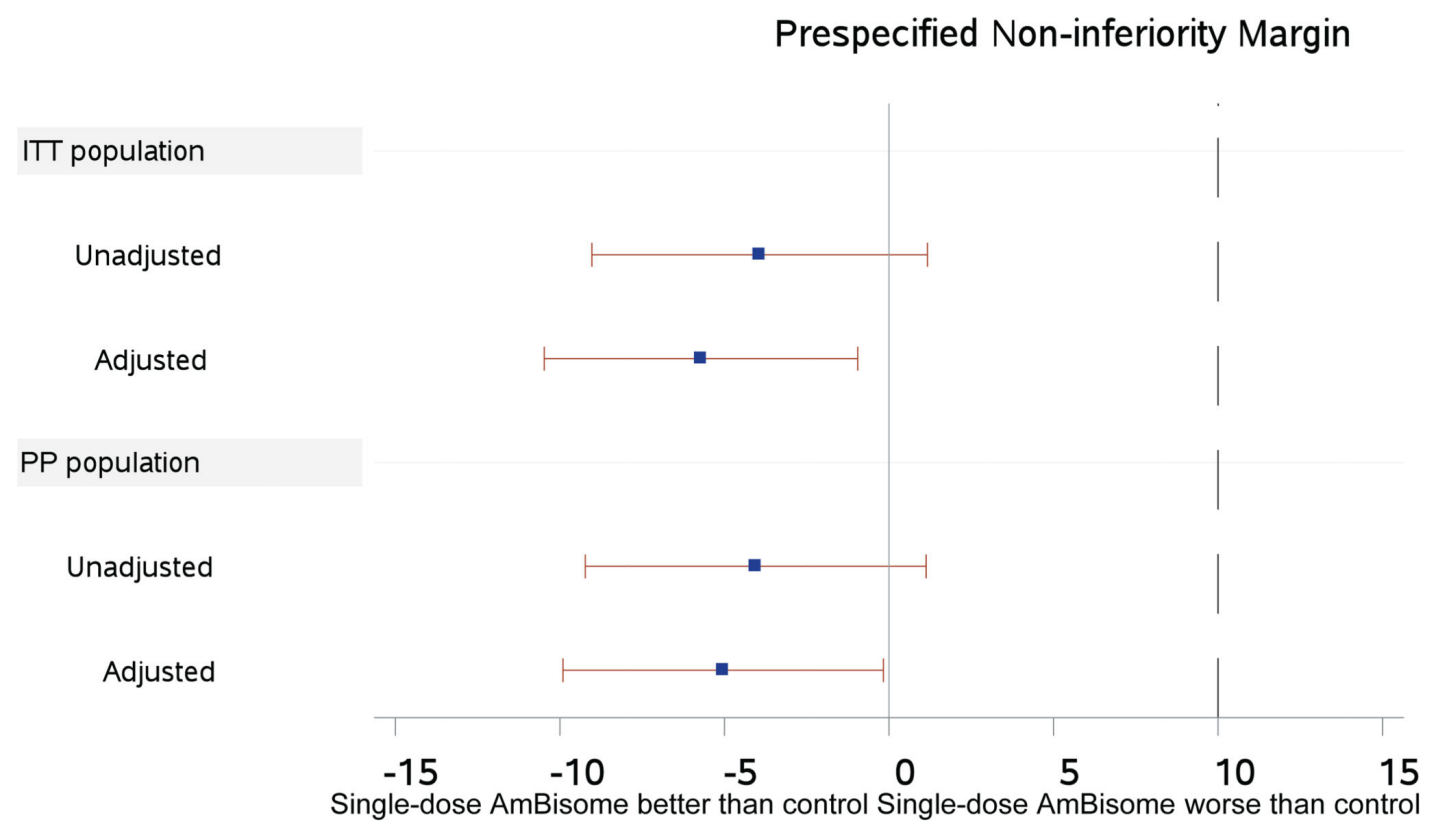
All-cause mortality. Panel A shows the cumulative all-cause mortality by week 10 according to treatment strategy in the intention-to-treat population. Panel B shows a non-inferiority graph for differences in all-cause mortality at 10 weeks. The mean difference in 10-week mortality risk (absolute percentage difference) between the AmBisome and control arms and the two-sided 90% confidence intervals in both unadjusted and adjusted intention-to-treat and per-protocol analyses are shown. The dashed line indicated the prespecified 10% non-inferiority margin. Adjusted analysis adjusting for pre-specified baseline covariates of: site, age, sex, Glasgow Coma Scale, CD4 count, cerebrospinal fluid colony forming units/mL, HIV therapy status, hemoglobin, and CSF opening pressure.

In the intention-to-treat analysis, the proportion who died at 10 weeks was 24.8% (101 of 407; 95% CI, 20.7-29.3%) in the AmBisome group compared with 28.7% (117 of 407; 95% CI, 24.4-33.4%) in the control group (Table 2 and Figure 2A). The absolute difference in 10-week mortality risk between the AmBisome arm and control arm was -3.9% and the upper limit of the one-sided 95% confidence interval for this mortality risk difference was 1.2%, within the pre-specified 10% non-inferiority margin (p-value for non-inferiority <0.001)(Figure 2B). In the per-protocol analysis, mortality risk at 10 weeks was 24.5% (95 of 388; 95% CI, 20.3-29.1%) in the AmBisome group compared with 28.5% (113 of 396; 95% CI, 24.1-33.3%) in the control group, with a 10-week mortality risk difference of -4.1%, and upper limit of one-sided 95% confidence interval 1.1%. Results were consistent across protocol defined adjusted analyses (Table 2, Figure 2B) and key sub-group analyses (Supplementary Appendix Table S3).

### Other mortality outcomes

Mortality risk at 2 weeks, 4 weeks, and 16 weeks is shown in Supplementary Appendix Table S3. The AmBisome group was non-inferior to the control group at the 10% margin at each of these time-points in both intention-to-treat and per-protocol populations. In a pre-specified superiority analysis at the 10-week time-point, the reduction in mortality risk in the AmBisome group compared with the control group did not reach statistical significance in the unadjusted analyses (risk difference -3.9%; 95% CI, -10.0% to 2.2%;), but was statistically significant in adjusted analysis (risk difference - 5.7%; 95% CI, -11.4% to -0.04%) when adjusting for co-variates associated with cryptococcal mortality. Outcomes of time-to-event analyses of mortality risk using Cox regression are shown in Supplementary Appendix Table S4, with no significant differences between groups (Figure 2B).

### Rate of clearance of *Cryptococcus* from the CSF

The mean rate of fungal clearance from the CSF was -0.40 log_10_ CFU/ml/day in the AmBisome group and -0.42 log_10_ CFU/ml/day in the control group, difference in mean rates of 0.017 log_10_ CFU/ml/day; (95% CI, -0.001 to 0.036)(Table 2B and Supplementary Appendix Figure S2).

### Immune reconstitution inflammatory syndrome, relapse, and hospital re-admission

Paradoxical IRIS was reported in 3.7% (15 of 407) participants in the AmBisome group and 4.7% (19 of 407) in the control group (Supplementary Appendix Table S6). There were no cases of culture-positive relapse in the AmBisome arm. One case of relapse occurred in a participant who had received full induction therapy within the control group and initially cleared *Cryptococcus* from the CSF but had subsequent poor adherence to consolidation phase fluconazole. Overall, during the initial 10 weeks of follow up 17.4% (71 of 407) of participants were re-admitted to hospital at least once in the AmBisome arm and 17.4% (71 of 407) in the control arm (Supplementary Appendix Table S7).

### Safety and adverse events

In the safety population, including all randomized participants who received one or more doses of study medication, within the initial 21 days of treatment there were 382 grade 3 or 4 adverse events among 210/420 (50.0%) of participants randomized into the AmBisome group and 579 grade 3 or 4 adverse events among 263/422 (62.3%) participants randomized into the control group (p<0.001). Table 3 gives a summary of clinical and laboratory adverse events, with a detailed listing in Supplementary Appendix Table S8. Potentially life threatening (grade 4) adverse events occurred in significantly fewer participants in the AmBisome group than the control group (21.7% [91 of 420] vs. 30.1% [127 of 422], p=0.005). Grade 3 or 4 anemia developed in 13.3% (56 of 420) of participants in the AmBisome group compared to 39.1% (165 of 422) in the control group (p<0.001). The mean decrease in hemoglobin over the first week of the induction period was 0.3g/dL in the AmBisome group and 1.9g/dL in the control group (p<0.001); 7.6% (32 of 420) of participants in the AmBisome group received a blood transfusion, compared to 18.0% (76 of 422) of participants in the control group. A grade 3 or 4 increase in creatinine developed in 5.2% (22 of 420) of participants in the AmBisome group compared to 5.9% (25 of 422) in the control group. The mean relative increase in serum creatinine from baseline to day 7 was 20.2% in the Ambisome group and 49.7% in the control group (p<0.001). Thrombophlebitis requiring antibiotic therapy occurred in 1.9% (8 of 420) of participants in the AmBisome group and 6.7% (28 of 422) of participants in the control group (p=0.001). There was a low frequency of grade 4 thrombocytopenia, neutropenia, and elevated alanine aminotransferase in both AmBisome and control groups.

## Discussion

This trial showed that induction therapy with a single 10 mg/kg dose of liposomal amphotericin B (AmBisome) with oral flucytosine and fluconazole was non-inferior to the WHO recommended standard-of-care of one week of amphotericin B deoxycholate given with flucytosine and was associated with significantly fewer adverse events. This clinical trial of cryptococcal meningitis was conducted in a range of settings across five countries in southern and eastern Africa with no loss to follow-up, giving generalizability to high HIV prevalent African settings (Supplementary Appendix Table S9).

The 10-week mortality of 24.8% seen in the AmBisome group of our trial is among the lowest reported from a major cryptococcal meningitis trial in Africa, despite more than a quarter of participants presenting with very severe disease and abnormal baseline mental status. Our trial demonstrates that both strategies of single-dose AmBisome and short-course amphotericin B deoxycholate treatment with flucytosine are capable of reducing 10-week mortality from cryptococcal meningitis to below 30%, a significant improvement on the rates of 40-45% reported with 2-week amphotericin B-based regimens in trials from resource limited settings,^12,29-31^ and is consistent with the relatively favorable outcomes with the 1-week amphotericin B deoxycholate plus flucytosine regimen that were reported in the ACTA trial.^12^

The trial builds on the phase-II data^24^ that a single 10mg/kg dose of AmBisome is effective at clearing *Cryptococcus* from the CSF. Single high-dose AmBisome with flucytosine and fluconazole matched the fungicidal activity of seven days of amphotericin B deoxycholate, at 1 mg/kg, plus flucytosine. In addition, the single high-dose AmBisome treatment regimen was better tolerated than the one-week amphotericin B deoxycholate regimen, with fewer adverse events overall, fewer life-threatening grade 4 events, fewer episodes of grade 3 or 4 anemia, a reduced need for blood transfusion, and less thrombophlebitis. This reflects the known improved drug toxicity-profile of liposomal amphotericin B compared to amphotericin B deoxycholate.^13,15^ Within this trial we administered pre-emptive fluid and electrolytes to all participants to reduce the risk of amphotericin B-related toxicity, adopted an intensive blood monitoring schedule, and actively managed adverse events when they occurred. The reality of routine care in resource-limited settings is that the necessary resources are often not available to implement this toxicity reduction and intensive monitoring and management approach.

A further potential benefit of the AmBisome regimen is that it may be possible to shorten the length of hospital stay needed to safely administer effective treatment. For the evaluation of safety in this trial, our protocol required all participants to be admitted to hospital for seven days of inpatient monitoring. However, when scaled-up in real-world situations, earlier discharge will likely be possible in a proportion of participants. A cost-effectiveness comparison is underway. Given our results, a single high dose liposomal amphotericin B approach may be worth investigating in the treatment of other systemic fungal infections prevalent in resource-limited settings, such as histoplasmosis and talaromycosis.^32,33^

Our trial was open label, and clinical management of the critically unwell participants with advanced HIV disease was complex. However, both the primary endpoint of all-cause mortality and the key safety endpoints of laboratory confirmed toxicities were objectively measured, and a consistent approach to HIV and ART management agreed by the investigators and applied throughout the study (see Supplementary Appendix Table S10), avoiding differential management or outcome assessment by study arm.

In conclusion, a single, high-dose of AmBisome given with flucytosine and fluconazole was non-inferior to the current WHO recommended standard of care for cryptococcal meningitis and offers a practical, easier-to-administer and better-tolerated treatment for the management for HIV-associated cryptococcal meningitis. Continued efforts to ensure access to AmBisome and flucytosine are needed to enable implementation of this treatment.

## Supplementary Material

Supplement

## Figures and Tables

**Table 1:** Baseline characteristics of the participants.

Characteristic	AmBisome (N=407)	Control (N=407)
Sex – no. (%) male	246 (60.4)	245 (60.2)
Median age (IQR) – years	37 (32-44)	37 (32-43)
New diagnosis of HIV – no. (%)	127 (31.2)	118 (29.0)
Reported prior antiretroviral therapy*– no. (%)	256 (62.9)	266 (65.4)
Median weight (IQR) – kg	53 (47-60)	53 (48-60)
Current headache – no. (%)	390 (95.8)	394 (96.8)
Median duration of headache (IQR) – days	14 (7-21)	14 (7-21)
Seizures in 72 hours before enrolment – no. (%)	45 (11.1)	42 (10.3)
Glasgow Coma Scale score <15 – no. (%)	115 (28.3)	117 (28.7)
Median CSF quantitative culture (IQR) – CFU/mL†	48,500 (300-420,000)	42,000 (585-365,000)
Median CSF opening pressure (IQR) – cm H**2**O‡	21 (14-32)	21 (13-31)
CSF opening pressure >25cm H**2**O– no. (%)‡	165 (41.4)	158 (39.5)
Median CSF white-cell count (IQR) – cells/mm^3^§	6 (4-75)	5 (3-52)
Median CSF glucose level (IQR) – mmol/L¶	2.5 (1.6-3.4)	2.4 (1.5-3.2)
Median CSF protein level (IQR) – g/L**	0.90 (0.46-1.48)	0.84 (0.44-1.38)
Median hemoglobin level (IQR) – g/dL††	11.2 (9.7-12.7)	11.2 (9.6-12.9)
Median creatinine level (IQR) – μmol/L‡‡	65 (53-80)	68 (55-86)
Median baseline CD4+ cell count (IQR) – cells/mm^3^§§	26 (9-56)	28 (11-59)

CFU denotes colony-forming units, CSF cerebrospinal fluid, HIV human immunodeficiency virus, and IQR interquartile range.

* Median time to ART re-initiation or switch in those with prior ART exposure was 30 in the AmBisome group and 29 days in the control group.

† Data were missing for 1 participant in the AmBisome group.

‡ Data were missed for 8 participants in the AmBisome group and 7 participants in the control group.

§ Data were missing for 11 participants in the AmBisome group and 9 participants in the control group.

¶ Data were missing for 11 participants in the AmBisome group and 15 participants in the control group.

** Data were missing for 14 participants in the AmBisome group and 16 participants in the control group.

†† Data were missing for 2 participants in the AmBisome group and 1 participants in the control group.

‡‡ Data were missing for 1 participant in the AmBisome group

§§ Data were missing for 18 participants in the AmBisome group and 11 participants in the control group.

**Table 2:** Primary outcome data. CI denotes confidence interval.

2A: Mortality data								
			Unadjusted Analysis			Adjusted Analysis*		
Outcome	AmBisome (N=407)	Control (N=407)	Risk Difference (%)	Upper bound of one-sided 95% CI (%)	95% CI (%)	Risk Difference (%)	Upper bound of one-sided 95% CI (%)	95% CI (%)
**Intention to treat population**								
**Mortality at 10 weeks**								
No. of deaths % (95% CI)	101 24.8 (20.7 to 29.3)	117 28.7 (24.4 to 33.4)	-3.93	1.2	-10.0 to 2.2	-5.71	-1.0	-11.4 to - 0.04
**Per-protocol population**								
	**(N=388)**	**(N=396)**						
**Mortality at 10 weeks**								
No. of deaths % (95% CI)	95 24.5 (20.3 to 29.1)	113 28.5 (24.1 to 33.3)	-4.05	1.1	-10.2 to 2.1	-5.04	-0.2	-10.8 to 0.8

* Adjusted analysis adjusting for pre-specified baseline covariates of: site, age, sex, Glasgow Coma Scale, CD4 count, cerebrospinal fluid colony forming units/mL, antiretroviral therapy status, hemoglobin, and CSF opening pressure.

**2B:** Early Fungicidal Activity – Intention to treat population

Outcome	AmBisome Arm	Control Arm	Difference (95% CI)	P value
**Mixed effects model** *				
**No. of participants**	363	381		
**Log_10_CFU/ml/day** **Mean (SD)**	-0.40 (0.13)	-0.42 (0.13)	0.017 (-0.001 to 0.036)	0.07

Participants needed a non-sterile cerebrospinal fluid culture at baseline to be included in this analysis.

* To enable comparison with prior published data using individual patient linear regression models to derive early fungicidal activity we also analyzed our data using linear regression. Early fungicidal activity was -0.41 (standard deviation 0.19) Log_10_CFU/ml/day in the Ambisome group and -0.44 (standard deviation 0.21) Log_10_CFU/ml/day in the Control group, p=0.12.

**Table 3:** Laboratory-Defined and Clinical Adverse Events Occurring within 21 Days of Randomization, According to Treatment Strategy.

Event	AmBisome (N=420)	Control (N=422)	P value*
Total number of Grade 3 or 4 adverse events	382	579	
Any adverse event – no. of participants (%)			
Grade 3 or 4	210 (50.0)	263 (62.3)	<0.001
Grade 3	173 (41.2)	225 (53.3)	<0.001
Grade 4	91 (21.7)	127 (30.1)	0.005
Anemia – no. of participants (%)			
Grade 3† (hemoglobin 7.0 to <9.0 g/ dL in men; 6.5 to <8.5 g/dL in women)	44 (10.5)	108 (25.6)	<0.001
Grade 4‡ (hemoglobin <7.0 g/dL in men; <6.5 g/dL in women)	12 (2.9)	62 (14.7)	<0.001
Mean change in hemoglobin level from baseline to day 7 (SD) – g/dl^†^	-0.3 (1.39)	-1.9 (1.8)	<0.001
Received a blood transfusion – no. of participants (%)	32 (7.6)	76 (18.0)	<0.001
Neutropenia – no. of participants (%)			
Grade 3† (neutrophils 400 to 599 per mm^3^)	27 (6.4)	21 (5.0)	0.36
Grade 4‡ (neutrophils <400 per mm^3^)	20 (4.8)	16 (3.8)	0.49
Thrombocytopenia– no. of participants (%)			
Grade 3† (thrombocytes 25,000 to 49,999 per mm^3^)	9 (2.1)	17 (4.0)	0.11
Grade 4‡ (thrombocytes <25,000 per mm^3^)	4 (1.0)	6 (1.4)	0.75
Creatinine increase – no. of participants (%)			
Grade 3† (2.47 to 4.42 mg/dL or 216 to 400 μmol/L)	17 (4.0)	22 (5.2)	0.42
Grade 4‡ (>4.42 mg/dL or >400 μmol/L)	5 (1.2)	3 (0.7)	0.51
Mean % change in creatinine level from baseline to day 7 (SD) ‡	20.2 (48.1)	49.7 (70.8)	<0.001
Hypokalaemia – no. of participants (%)			
Grade 3† (potassium 2.0 to 2.4 mmol/L)	6 (1.4)	27 (6.4)	<0.001
Grade 4‡ (potassium < 2.0 mmol/L)	0 (0.0)	3 (0.7)	0.25
Elevated ALT – no. of participants (%)			
Grade 3† (ALT 200 to 400 IU/L)	6 (1.4)	4 (0.9)	0.52
Grade 4‡ (ALT >400 IU/L)	1 (0.2)	1 (0.2)	1.0
Thrombophlebitis requiring antibiotic therapy - no. of participants (%)	8 (1.9)	28 (6.6)	<0.001
Other Grade 3 or 4 adverse event^$^ - no. of participants (%)	167 (39.8)	173 (41.0)	0.72

The adverse event data are presented for the safety population, consisting of all randomized participants who received who received one or more doses of study medication. One participant in the AmBisome group withdrew consent after randomization before receiving any study treatment and one participant in the control group died after randomization but before receiving any study treatment. Both were excluded from the safety analysis.

* P-values derived from chi-squared or t-tests as appropriate.

† Data were only available for participants with baseline and day 7 values, missing in 50 participants in the AmBisome group and 61 participants in the control group.

‡ Data were only available for participants with baseline and day 7 values, missing for 42 participants in the AmBisome group and 50 participants in the control group.

$ During the course of the trial there were two infusion reactions that met the grade 3 criteria, both in participants receiving AmBisome. Both responded to simple supportive measures. There were no study participants in whom the prescribed dose of either AmBisome or amphotericin B deoxycholate could not be given due to infusion side effects. We did not collate data on milder infusion reactions.
