# Supplementary material for: Single-Dose Liposomal Amphotericin B Treatment for Cryptococcal Meningitis
Source: N Engl J Med. Author manuscript; Available in PMC 2022 Apr 28. (PMC7612678; doi:10.1056/NEJMoa2111904)
Supplement: Supplement [file EMS143842-supplement-Supplement.pdf]

SUPPLEMENTARY APPENDIX

TABLE OF CONTENTS

THE AMBITION STUDY GROUP 2

FIGURE S1: FOREST PLOT SHOWING PERCENTAGE ABSOLUTE RISK DIFFERENCE IN 10-WEEK MORTALITY BETWEEN AMBISOME GROUP AND CONTROL GROUP BY PROTOCOL DEFINED SUBGROUP. 3

FIGURE S2: RATE OF CEREBROSPINAL FLUID FUNGAL CLEARANCE ACCORDING TO TREATMENT STRATEGY IN THE INTENTION-TO-TREAT POPULATION. 4

TABLE S1: STUDY MONITORING SCHEDULE 5

TABLE S2: STUDY EXCLUSIONS 6

TABLE S3: MORTALITY DATA 7

TABLE S3(A): MORTALITY DATA – SECONDARY OUTCOMES........ 7

TABLE S3(B): SUBGROUP ANALYSIS OF ALL-CAUSE MORTALITY WITHIN THE INTENTION TO TREAT POPULATION. 8

TABLE S4: TIME TO EVENT ANALYSIS WITHIN THE INTENTION TO TREAT POPULATION. 9

TABLE S5: DISABILITY AND MODIFIED RANKIN SCORE OUTCOMES IN THE INTENTION TO TREAT POPULATION 10

TABLE S6: LINE LISTING OF IMMUNE RECONSTITUTION INFLAMMATORY SYNDROME (IRIS) CASES 11

TABLE S7: SUMMARY AND LINE LISTING OF READMISSIONS DURING THE STUDY PERIOD 13

TABLE S8: ADDITIONAL DATA RELATED TO CLINICAL AND LABORATORY DEFINED ADVERSE EVENTS. 17

TABLE S9: SUPPLEMENTARY TABLE ON THE REPRESENTATIVENESS OF STUDY PARTICIPANTS. 18

TABLE S10: ANTIRETROVIRAL THERAPY MANAGEMENT SOP 20

The Ambition Study Group

In addition to the named authors, the following were members of the Ambition Study Group:

Botswana Harvard AIDS Institute Partnership / Princess Marina Hospital, Gaborone, Botswana – J Goodall, K Lechiile, N Mawoko, T Mbangiwa, J Milburn, R Mmipi, C Muthoga, P Ponatshego, I Rulaganyang, K Seatla, N Tlhako and K Tsholo.

University of Cape Town / Mitchells Plain Hospital / Khayelitsha District Hospital, Cape Town, South Africa – S April, A Bekiswa, L Boloko, H Bookholane, T Crede, L Davids, R Goliath, S Hlungulu, R Hoffman, H Kyepa, N Masina, D Maughan, T Mnguni, S Moosa, T Morar, M Mpalali, J Naude, I Oliphant, S Sayed, L Sebesho, M Shey and L Swanepoel.

Malawi-Liverpool-Wellcome Trust Clinical Research Programme / Queen Elizabeth Central Hospital, Blantyre, Malawi – M Chasweka, W Chimang'anga, T Chimphambano, E Dziwani, E Gondwe, A Kadzilimbile, S Kateta, E Kossam, C Kukacha, B Lipenga, J Ndaferankhande, M Ndalama, R Shah, A Singini, K Stott and A Zambasa.

UNC Project, Kamuzu Central Hospital, Lilongwe, Malawi – T Banda, T Chikaonda, G Chitulo, L Chiwoko, N Chome, M Gwin, T Kachitosi, B Kamanga, M Kazembe, E Kumwenda, M Kumwenda, C Maya, W Mhango, C Mphande, L Msumba, T Munthali, D Ngoma, S Nicholas, L Simwinga, A Stambuli, G Tegha and J Zambezi.

Infectious Diseases Institute / Kiruddu General Hospital, Kampala, Uganda – C Ahimbisibwe, A Akampurira, A Alice, F Cresswell, J Gakuru, D Kiiza, J Kisembo, R Kwizera, F Kugonza, E Laker, T Luggya, A Lule, A Musubire, R Muyise, O Namujju, J Ndyetukira, L Nsangi, M Okirwoth, A Sadiq, K Tadeo, A Tukundane and D Williams.

Infectious Diseases Institute / Mbarara Regional Referral Hospital, Mbarara, Uganda – L Atwine, P Buzaare, M Collins, N Emily, C Inyakuwa, S Kariisa, J Mwesigye, S Niwamanya, A Rodgers, J Rukundo, I Rwomushana, M Ssemusu and G Stead.

University of Zimbabwe / Parirenyatwa General Hospital, Harare, Zimbabwe – K Boyd, S Gondo, P Kufa, E Makaha, C Moyo, T Mtisi, S Mudzingwa, T Mwarumba and T Zinyandu.

Institut Pasteur, Paris, France – Alexandre Alanio, Françoise Dromer and Aude Sturny-Leclere.

London School of Hygiene and Tropical Medicine, London, UK – P Griffin and S Hafeez.

Figure S1: Forest plot showing percentage absolute risk difference in 10-week mortality between AmBisome group and Control group by protocol defined subgroup. ART denotes antiretroviral therapy, CSF cerebrospinal fluid, and GCS Glasgow Coma Scale. Colony forming units (CFUs) are reported in CFU/ml.

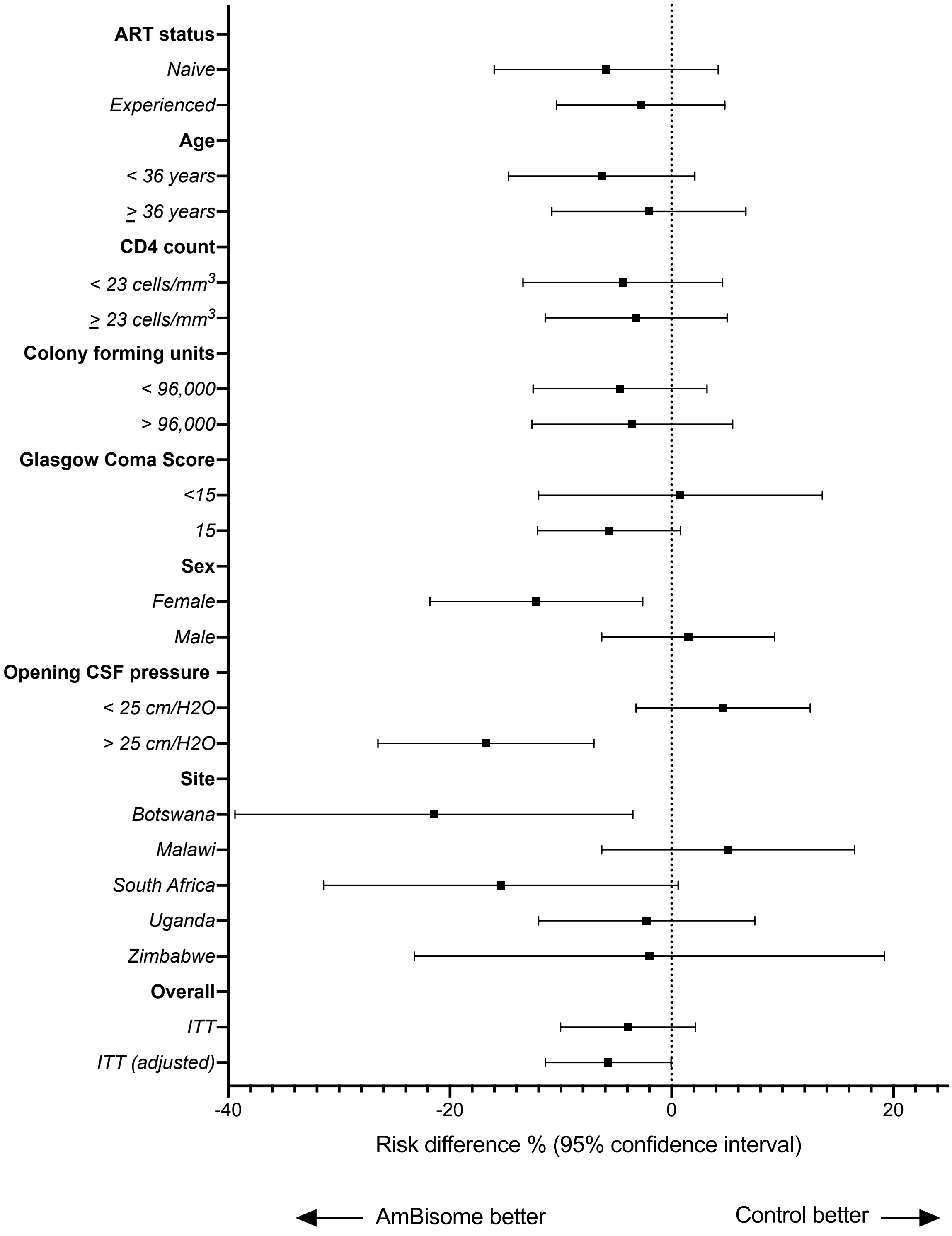

Figure S2: Rate of cerebrospinal fluid fungal clearance according to treatment strategy in the intention-to-treat population. The mean slope (standard deviation) is given below each plot. CFU denotes colony-forming units.

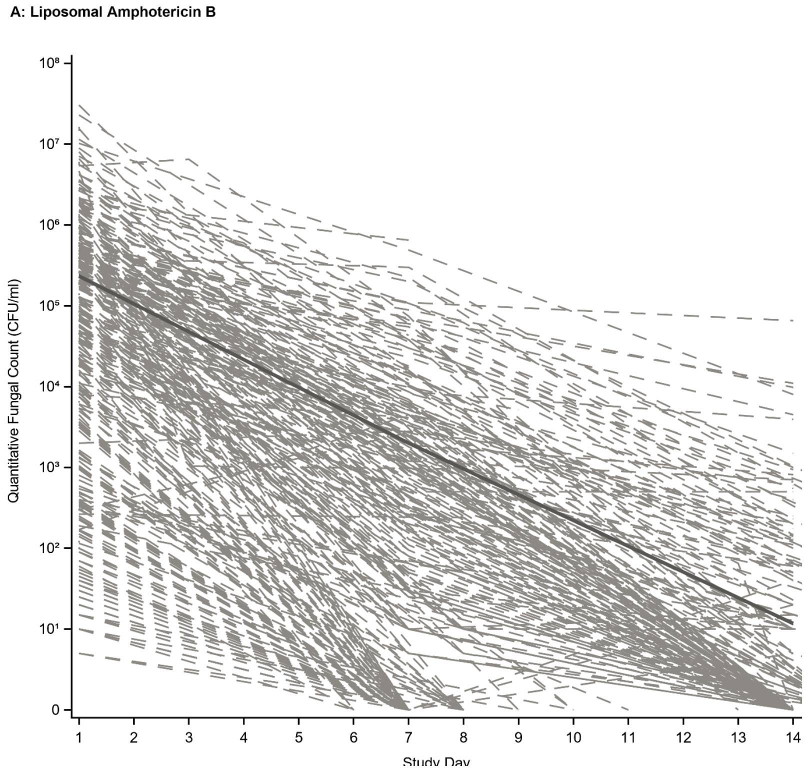

Mean rate of fungal clearance -0.40 $\log_{10}$ CFU/ml/day
(standard deviation 0.13 $\log_{10}$ CFU/ml/day)

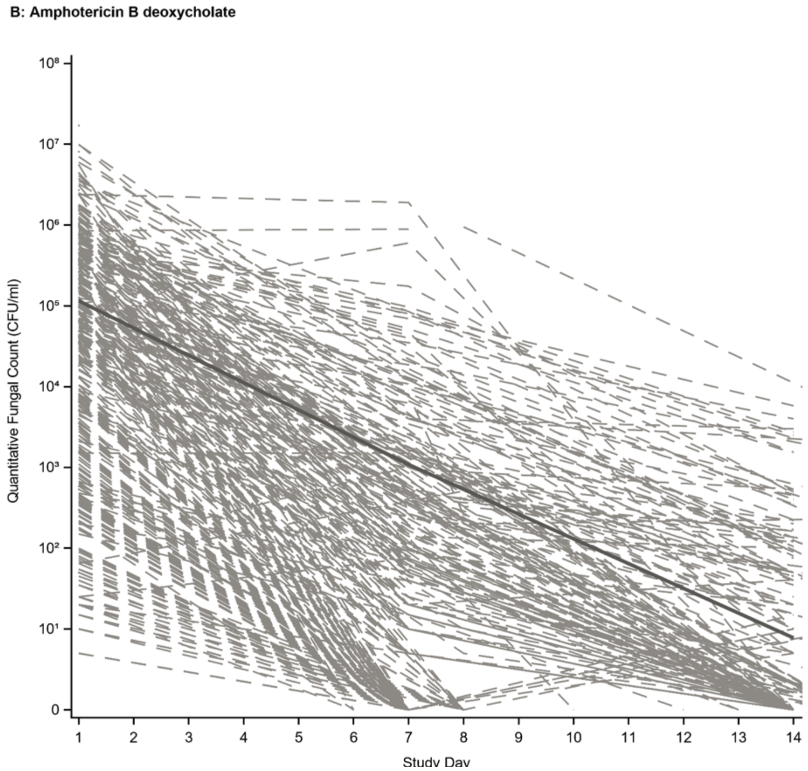

Mean rate of fungal clearance -0.42 $\log_{10}$ CFU/ml/day
(standard deviation 0.13 $\log_{10}$ CFU/ml/day)

Table S1: Study monitoring schedule*

	Screening	Week 1							Week 2							Wk 4	Wk 6	Wk 8	Wk 10	Wk 16
**Study Day**	≤D0	D1	D2	D3	D4	D5	D6	D7	D8	D9	D10	D11	D12	D13	D14					
**Consent forms**																				
PIS and signed consent	X	X																		
**Follow up**																				
Screening and randomisation	X	X																		
Clinical review		X	X	X	X	X	X	X	X	X	X	X	X	X	X					
Outpatient follow-up																X	X	X	X	
Week 16 telephone																				X
**Clinical labs**																				
HIV testing		X																		
Pregnancy Test (Urine/Serum)§		X																		
Full Blood Count		X						X							X	X				
CD4 count		X																		
ALT		X						X							X	X				
Urea, creatinine and electrolytes		X		X		X		X			X		X		X	X				
**Clinical evaluation**																				
Chest X-ray†		X																		
**CSF**																				
Opening pressure		X						X							X					
Cell count and differential‡		X																		
Protein, glucose‡		X																		
Routine culture ‡		X																		
India ink examination‡¶		X																		
Cryptococcal antigen‡¶		X																		
Quantitative fungal culture		X						X							X					
CSF Drug levels		X						X							X					
Immune parameters		X						X							X					

*ALT denotes alanine aminotransferase, CSF cerebrospinal fluid, HIV human immunodeficiency virus, and PIS participant information sheet. § For women of childbearing age, † If clinically indicated, ‡ Part of routine care, ¶ India ink or cryptococcal antigen required for inclusion.

Table S2: Study exclusions

Category	AmBisome	Control
**Included in error (n=6):**		
Did not have cryptococcal meningitis	1	4
Did not have HIV	1	0
**Late exclusions (n=24)*:**		
Alanine transaminase >5 times the upper limit of normal (>200 IU/L)	2	1
Polymorphonuclear leukocytes <500 x 106/L	3	5
Platelets <50,000 x 106/L	9	8
*The total exceeds 24 as 3 prticipants had bothe low platelets and low neutrophils, and one participant had both low platelets and a raised ALT.		
**Excluded from per-protocol analysis (n=30):**		
Missed more than 1 day of any single treatment in the first 2 weeks	15	5
Missed more than 2 weeks of fluconazole consolidation treatment between weeks 2 and 10	1	3
Incorrect induction received	3	3

Table S3: Mortality data

Table S3(A): Mortality data – secondary outcomes

			Unadjusted analysis			Adjusted analysis*		
**Outcome**	**AmBisome (N=407)**	**Control (N=407)**	**Risk Difference (%)**	**Upper bound of one-sided 95% CI (%)**	**95% CI (%)**	**Risk Difference (%)**	**Upper bound of one-sided 95% CI (%)**	**95% CI (%)**
**Intention to treat population – secondary outcomes**								
**Mortality at 2 weeks**								
No. of deaths	53	51	0.49	4.3	-4.1 to 5.1	-0.45	4.3	-6.1 to 5.2
% (95% CI)	13.0 (9.9 to 16.7)	12.5 (9.5 to 16.1)						
**Mortality at 4 weeks**								
No. of deaths	70	76	-1.47	3.0	-6.7 to 3.8	-1.27	3.4	-6.8 to 4.3
% (95% CI)	17.2 (13.7 to 21.1)	18.7 (15.0 to 22.8)						
**Mortality at 16 weeks**								
No. of deaths	115	119	-0.98	4.2	-7.2 to 5.2	-2.32	2.6	-8.2 to 3.5
% (95% CI)	28.3 (23.9 to 32.9)	29.2 (24.9 to 33.9)						
**Per-protocol population – secondary outcomes**								
**Mortality at 2 weeks**								
No. of deaths	51	50	0.52	4.5	-4.2 to 5.2	-0.11	5.0	-6.2 to 6.0
% (95% CI)	13.1 (10.0 to 16.9)	12.6 (9.5 to 16.3)						
**Mortality at 4 weeks**								
No. of deaths	66	74	-1.68	2.8	-7.0 to 3.7	-1.40	3.8	-7.6 to 4.8
% (95% CI)	17.0 (13.4 to 21.1)	18.7 (15.0 to 22.9)						
**Mortality at 16 weeks**								
No. of deaths	108	115	-1.21	4.1	-7.5 to 5.1	-2.36	2.6	-8.3 to 3.6
% (95% CI)	27.8 (23.4 to 32.6)	29.0 (24.6 to 33.8)						

*Adjusted analysis adjusting for pre-specified baseline covariates of: site, age, sex, Glasgow Coma Scale, CD4 count, cerebrospinal fluid colony forming units/mL, antiretroviral therapy status, hemoglobin, and CSF opening pressure.

Table S3(B): Subgroup analysis of all-cause mortality within the intention to treat population. *ART denotes antiretroviral therapy, CSF cerebrospinal fluid, and GCS Glasgow Coma Scale.

**Subgroup**	**Mortality at 10 weeks** *No. of deaths / No of patients (%)*		Risk Difference (%)	95% confidence interval (%)
	**AmBisome**	**Control**		
**ART status**				
**Naive**	35/151 (23.2)	41/141 (29.1)	-5.90	-16.0 to 4.2
**Experienced**	66/256 (25.8)	76/266 (28.6)	-2.79	-10.4 to 4.8
**Age**				
**< 36 years**	39/191 (20.4)	54/202 (26.7)	-6.31	-14.7 to 2.1
**≥ 36 years**	62/216 (28.7)	63/205 (30.7)	-2.03	-10.8 to 6.7
**CD4 count**				
**< 23 cells/mm^3**	56/203 (27.6)	63/197 (32.0)	-4.39	-13.4 to 4.6
**≥ 23 cells/mm^3**	37/186 (19.9)	46/199 (23.1)	-3.22	-11.4 to 5.0
**Colony forming units**				
**< 96,000**	36/197 (18.3)	47/205 (22.9)	-4.65	-12.5 to 3.2
**≥ 96,000**	65/209 (31.1)	70/202 (34.7)	-3.55	-12.6 to 5.5
**GCS**				
**<15**	52/115 (45.2)	52/117 (44.4)	0.77	-12.0 to 13.6
**15**	49/292 (16.8)	65/290 (22.4)	-5.63	-12.1 to 0.8
**Sex**				
**Female**	34/161 (21.1)	54/162 (33.3)	-12.22	-21.8 to -2.6
**Male**	67/246 (27.2)	63/245 (25.7)	1.52	-6.3 to 9.3
**Opening CSF pressure**				
**< 25 cm/H_2O**	65/234 (27.8)	56/242 (23.1)	4.64	-3.2 to 12.5
**≥ 25 cm/H_2O**	34/165 (20.6)	59/158 (37.3)	-16.74	-26.5 to -7.0
**Site**				
**Botswana**	6/42 (14.3)	15/42 (35.7)	-21.43	-39.4 to -3.5
**Malawi**	32/113 (28.3)	26/112 (23.2)	5.10	-6.3 to 16.5
**South Africa**	9/54 (16.7)	17/53 (32.1)	-15.41	-31.4 to 0.6
**Uganda**	44/163 (27.0)	48/164 (29.3)	-2.27	-12.0 to 7.5
**Zimbabwe**	10/35 (28.6)	11/36 (30.6)	-1.98	-23.2 to 19.2

Table S4: Time to event analysis within the intention to treat population. *CI denotes confidence interval.

Outcome		AmBisome (N=407)	Control (N=407)	Overall (N=814)
**2-week mortality**	No of events	53	51	104
	Person-years	14.3	14.2	28.5
	Incidence Rate per 100 person-years	370.18	359.03	364.63
	Hazard Ratio	1.03	Reference	
	95% CI of Hazard Ratio	0.70 to 1.52	Reference	
**4-week mortality**	No of events	70	76	146
	Person-years	27.5	27.3	54.8
	Incidence Rate per 100 person-years	254.62	278.16	266.36
	Hazard Ratio	0.92	Reference	
	95% CI of Hazard Ratio	0.66 to 1.27	Reference	
**10-week mortality**	No of events	101	117	218
	Person-years	63.6	62.3	125.9
	Incidence Rate per 100 person-years	158.88	187.73	173.17
	Hazard Ratio	0.85	Reference	
	95% CI of Hazard Ratio	0.65 to 1.11	Reference	
**16-week mortality**	No of events	115	119	234
	Person-years	101.8	98.8	200.6
	Incidence Rate per 100 person-years	112.96	120.47	116.66
	Hazard Ratio	0.95	Reference	
	95% CI of Hazard Ratio	0.73 to 1.22	Reference	

Table S5: Disability and Modified Rankin score outcomes in the intention to treat population

Description	Visit	Statistics	AmBisome (N=407)	Control (N=407)	Overall (N=814)
**Does the participant require help from anybody for everyday activities?**	Week 10	NO	266(65.4%)	250(61.4%)	516(63.4%)
		YES	36(8.8%)	35(8.6%)	71(8.7%)
		DIED	101(24.8%)	117(28.7%)	218(26.8%)
		NOT DONE	4(1.0%)	5(1.2%)	9(1.1%)
	Week 16	NO	278(68.3%)	269(66.1%)	547(67.2%)
		YES	13(3.2%)	18(4.4%)	31(3.8%)
		DIED	115(28.3%)	119(29.2%)	234(28.7%)
		NOT DONE	1(0.2%)	1(0.2%)	2(0.2%)
**Modified Rankin score**	Week 10	0	195(47.9%)	181(44.5%)	376(46.2%)
		1	55(13.5%)	59(14.5%)	114(14.0%)
		2	19(4.7%)	20(4.9%)	39(4.8%)
		3	18(4.4%)	13(3.2%)	31(3.8%)
		4	9(2.2%)	10(2.5%)	19(2.3%)
		5	6(1.5%)	3(0.7%)	9(1.1%)
		6	101(24.8%)	117(28.7%)	218(26.8%)
		NOT DONE	4(1.0%)	4(1.0%)	8(1.0%)
	Week 16	0	234(57.5%)	223(54.8%)	457(56.1%)
		1	38(9.3%)	40(9.8%)	78(9.5%)
		2	10(2.5%)	8(2.0%)	18(2.2%)
		3	7(1.7%)	9(2.2%)	16(2.0%)
		4	2(0.5%)	5(1.2%)	7(0.9%)
		5	0(0.0%)	2(0.5%)	2(0.2%)
		6	115(28.3%)	119(29.2%)	234(28.7%)
		NOT DONE	1(0.2%)	1(0.2%)	2(0.2%)

Table S6: Line listing of immune reconstitution inflammatory syndrome (IRIS) cases

Case	Treatment Group	Age (years)	Sex	Baseline CD4 count (cells/µL)	Baseline CSF WCC (cells/mm³)	Baseline CFUs (CFU/ml)	Day 14 CFUs (CFU/ml)	ART status	Study day of ART initiation	Study day on IRIS symptom onset	Number of days on ART at IRIS onset	CFU count at time of IRIS (CFU/ml)	Week Ten Outcome
**1**	Control	30	Female	3	5	44000	0	Switch	29	43	15	0	Alive
**2**	Control	38	Male	14	0	195000	45	Re-initiation	28	38	11	0	Alive
**3**	Control	44	Male	6	2	3490000	500	Initiation	28	42	15	0	Alive
**4**	Control	38	Female	106	264	130000	5	Switch	30	63	34	ND	Dead
**5**	Control	41	Male	23	<5	1800000	230	Switch	29	66	38	ND	Alive
**6**	Control	34	Male	6	<5	57000	0	Initiation	28	59	32	0	Alive
**7**	Control	35	Male	11	<5	4900	0	Switch	45	70	26	0	Alive
**8**	Control	49	Male	53	<5	665	0	Initiation	33	63	31	0	Dead
**9**	Control	44	Male	7	110	38500	0	Initiation	27	43	17	0	Alive
**10**	Control	41	Male	20	<5	500000	20	Initiation	29	57	29	0	Alive
**11**	Control	56	Male	171	155	0	0	Switch	43	61	19	0	Dead
**12**	Control	32	Female	6	<5	960000	10	Initiation	16	36	21	0	Dead
**13**	Control	36	Male	27	<5	700000	200	Switch	29	38	10	0	Alive
**14**	Control	20	Male	8	<5	50000	0	Switch	29	34	6	0	Alive
**15**	Control	31	Male	51	<5	350000	0	Re-initiation	43	64	22	0	Alive
**16**	Control	22	Female	56	<5	7500	0	Re-initiation	28	52	25	0	Alive
**17**	Control	41	Male	20	<5	415000	225	Initiation	35	70	36	ND	Alive
**18**	Control	45	Male	60	38	10000000	3000	Initiation	28	54	27	0	Alive
**19**	Control	44	Female	35	0	6000000	400	Initiation	28	35	8	60	Alive
**20**	AmBisome	45	Male	38	3	560000	940	Initiation	30	41	12	0	Dead
**21**	AmBisome	42	Male	6	2	305000	0	Initiation	30	43	14	0	Alive
**22**	AmBisome	45	Male	30	2	1265000	0	Switch	29	55	27	0	Alive

**23**	AmBisome	36	Female	8	1	350000	0	Switch	28	42	15	0	Alive
**24**	AmBisome	25	Female	5	1	310000	0	Continued*	1	31	31	0	Alive
**25**	AmBisome	47	Male	9	0	485000	45	Switch	28	39	12	0	Alive
**26**	AmBisome	43	Male	8	11	46500	0	Switch	43	57	15	ND	Alive
**27**	AmBisome	42	Male	50	100	190000	0	Re-initiation	28	37	10	0	Alive
**28**	AmBisome	33	Female	39	<5	175000	40	Switch	43	57	15	0	Alive
**29**	AmBisome	35	Female	2	<5	200000	200	Initiation	30	43	14	0	Alive
**30**	AmBisome	40	Female	20	155	22000	10	Re-initiation	28	46	19	0	Alive
**31**	AmBisome	40	Male	9	<5	70000	175	Re-initiation	29	63	35	ND	Dead
**32**	AmBisome	23	Male	12	<5	37000	0	Continued*	1	19	19	0	Alive
**33**	AmBisome	35	Male	7	<5	580000	3950	Initiation	25	53	29	90	Alive
**34**	AmBisome	32	Female	6	<5	350000	0	Initiation	42	49	8	0	Alive

WCC: white cell count; CFU: colony forming units; ART: antiretroviral therapy; ND: not done.

* Defaulter continued erroneously on ART at baseline

Immune reconstitution inflammatory syndrome was diagnosed according to the following case definition: Recurrence of symptoms of cryptococcal meningitis after initiating, re-initiating or switching antiretroviral therapy in the absence of an increase in cerebrospinal fluid quantitative cryptococcal culture.

Table S7: Summary and line listing of readmissions during the study period

**Summary of readmissions during study follow-up:**			
	**All**	**AmBisome**	**Control**
Directly related to the initial cryptococcal meningitis	57	27	30
Non-cryptococcal meningitis related	85	44	41
All	*142*	*71*	*71*

**Line listings of readmissions during study follow-up:**			
**Case**	**Treatment group**	**Reason**	**Cryptococcal meningitis related**
1	Control	Acute kidney injury	No
2	Control	Acute kidney injury	No
3	Control	Advanced HIV Disease	No
4	Control	Advanced HIV Disease	No
5	Control	Advanced HIV Disease	No
6	Control	Advanced HIV Disease	No
7	Control	Advanced HIV Disease	No
8	Control	Advanced HIV Disease	No
9	Control	Anaemia	No
10	Control	Anaemia	No
11	Control	Dehydration	No
12	Control	Deep vein thrombosis	No
13	Control	Deep vein thrombosis	No
14	Control	Electrolyte imbalance	No
15	Control	Gastroenteritis	No
16	Control	Idiopathic thrombocytopenia purpura	No
17	Control	Malaria	No
18	Control	Nasopharyngeal malignancy	No
19	Control	Pneumonia	No
20	Control	Pneumonia	No
21	Control	Psychosis	No
22	Control	Renal impairment	No
23	Control	Sepsis	No
24	Control	Sepsis	No
25	Control	Sepsis	No
26	Control	Sepsis	No
27	Control	Sepsis	No
28	Control	Sepsis	No
29	Control	Sepsis	No
30	Control	Sepsis	No
31	Control	Sepsis	No
32	Control	Sepsis	No
33	Control	Sepsis	No
34	Control	Sepsis	No
35	Control	Tuberculosis	No

36	Control	Tuberculosis	No
37	Control	Tuberculosis	No
38	Control	Tuberculosis (meningitis)	No
39	Control	Thrombophlebitis	No
40	Control	Urinary retention	No
41	Control	Warfarin Toxicity	No
42	Control	Paradoxical IRIS	Yes
43	Control	Paradoxical IRIS	Yes
44	Control	Paradoxical IRIS	Yes
45	Control	Paradoxical IRIS	Yes
46	Control	Paradoxical IRIS	Yes
47	Control	Paradoxical IRIS	Yes
48	Control	Paradoxical IRIS	Yes
49	Control	Paradoxical IRIS	Yes
50	Control	Paradoxical IRIS	Yes
51	Control	Paradoxical IRIS	Yes
52	Control	Persistent cryptococcal meningitis	Yes
53	Control	Persistent cryptococcal meningitis	Yes
54	Control	Persistent cryptococcal meningitis	Yes
55	Control	Persistent cryptococcal meningitis	Yes
56	Control	Persistent cryptococcal meningitis	Yes
57	Control	Persistent cryptococcal meningitis	Yes
58	Control	Persistent cryptococcal meningitis	Yes
59	Control	Persistent cryptococcal meningitis	Yes
60	Control	Persistent cryptococcal meningitis	Yes
61	Control	Persistent cryptococcal meningitis	Yes
62	Control	Persistent cryptococcal meningitis	Yes
63	Control	Persistent cryptococcal meningitis	Yes
64	Control	Persistent cryptococcal meningitis	Yes
65	Control	Persistent cryptococcal meningitis	Yes
66	Control	Persistent cryptococcal meningitis	Yes
67	Control	Persistent cryptococcal meningitis	Yes
68	Control	Persistent cryptococcal meningitis	Yes
69	Control	Persistent cryptococcal meningitis	Yes
70	Control	Persistent cryptococcal meningitis	Yes
71	Control	Persistent cryptococcal meningitis	Yes
72	AmBisome	Advanced HIV Disease	No
73	AmBisome	Advanced HIV Disease	No
74	AmBisome	Advanced HIV Disease	No
75	AmBisome	Advanced HIV Disease	No
76	AmBisome	Alcohol withdrawal	No
77	AmBisome	Anaemia	No
78	AmBisome	ART toxicity	No
79	AmBisome	Dehydration	No
80	AmBisome	Empyema	No
81	AmBisome	Gastroenteritis	No
82	AmBisome	Gastroenteritis	No
83	AmBisome	Gastroenteritis	No
84	AmBisome	Gastroenteritis	No
85	AmBisome	Gastroenteritis	No

86	AmBisome	Gastroenteritis	No
87	AmBisome	Gastroenteritis	No
88	AmBisome	Gastroenteritis	No
89	AmBisome	Gastroenteritis	No
90	AmBisome	Haemoltyic uraemic syndrome	No
91	AmBisome	Hyperkalaemia	No
92	AmBisome	Hyponatraemia	No
93	AmBisome	Kaposi's sarcoma	No
94	AmBisome	Malaria	No
95	AmBisome	Pneumonia	No
96	AmBisome	Pneumonia	No
97	AmBisome	Pneumonia	No
98	AmBisome	Psychosis	No
99	AmBisome	Sepsis	No
100	AmBisome	Sepsis	No
101	AmBisome	Sepsis	No
102	AmBisome	Sepsis	No
103	AmBisome	Sepsis	No
104	AmBisome	Sepsis	No
105	AmBisome	Sepsis	No
106	AmBisome	Severe malnutrition	No
107	AmBisome	Severe Zoster	No
108	AmBisome	Tuberculosis	No
109	AmBisome	Tuberculosis	No
110	AmBisome	Tuberculosis	No
111	AmBisome	Tuberculosis	No
112	AmBisome	Toxoplasmosis	No
113	AmBisome	Urinary tract infection	No
114	AmBisome	Urinary tract infection	No
115	AmBisome	Viral upper respiratory tract infection	No
116	AmBisome	Paradoxical IRIS	Yes
117	AmBisome	Paradoxical IRIS	Yes
118	AmBisome	Paradoxical IRIS	Yes
119	AmBisome	Paradoxical IRIS	Yes
120	AmBisome	Paradoxical IRIS	Yes
121	AmBisome	Paradoxical IRIS	Yes
122	AmBisome	Paradoxical IRIS	Yes
123	AmBisome	Paradoxical IRIS	Yes
124	AmBisome	Persistent cryptococcal meningitis	Yes
125	AmBisome	Persistent cryptococcal meningitis	Yes
126	AmBisome	Persistent cryptococcal meningitis	Yes
127	AmBisome	Persistent cryptococcal meningitis	Yes
128	AmBisome	Persistent cryptococcal meningitis	Yes
129	AmBisome	Persistent cryptococcal meningitis	Yes
130	AmBisome	Persistent cryptococcal meningitis	Yes
131	AmBisome	Persistent cryptococcal meningitis	Yes
132	AmBisome	Persistent cryptococcal meningitis	Yes
133	AmBisome	Persistent cryptococcal meningitis	Yes
134	AmBisome	Persistent cryptococcal meningitis	Yes
135	AmBisome	Persistent cryptococcal meningitis	Yes

136	AmBisome	Persistent cryptococcal meningitis	Yes
137	AmBisome	Persistent cryptococcal meningitis	Yes
138	AmBisome	Persistent cryptococcal meningitis	Yes
139	AmBisome	Persistent cryptococcal meningitis	Yes
140	AmBisome	Persistent cryptococcal meningitis	Yes
141	AmBisome	Seizures	Yes
142	AmBisome	Stroke	Yes

Table S8: Additional data related to clinical and laboratory defined adverse events. *SUSAR denotes suspected unexpected serious adverse reaction.

Event	AmBisome (N=420)	Control (N=422)	P value
Total number of SUSAR	0	0	
Study medication-related adverse event (possible) – no. of participants (%)			
Grade 3 or 4	75 (17.9)	104 (24.6)	0.016
Grade 3	59 (14.0)	79 (18.7)	0.067
Grade 4	22 (5.2)	35 (8.3)	0.078
Study medication-related adverse event (probable) – no. of participants (%)			
Grade 3 or 4	68 (16.2)	147 (34.8)	<0.001
Grade 3	57 (13.6)	121 (28.7)	<0.001
Grade 4	16 (3.8)	48 (11.4)	<0.001
Study medication-related adverse event (definite) – no. of participants (%)			
Grade 3 or 4	16 (3.8)	77 (18.2)	<0.001
Grade 3	10 (2.4)	61 (14.5)	<0.001
Grade 4	7 (1.7)	24 (5.7)	0.002
Study medication-related adverse event (possible, probable or definite) – no. of participants (%)			
Grade 3 or 4	115 (27.4)	219 (51.9)	<0.001
Grade 3	98 (23.3)	186 (44.1)	<0.001
Grade 4	36 (8.6)	80 (19.0)	<0.001
Mean change in hemoglobin level to day 14 (SD) – g/dl§	-0.2 (1.4)	-1.5 (2.1)	<0.001
Mean % change in creatinine level to day 14 (SD) ¶	20.2 (49.3)	36.6 (63.3)	<0.001

§Data were missing for 72 participants in the AmBisome group and 75 participants in the control group.

¶Data were missing for 62 participants in the AmBisome group and 65 participants in the control group.

Table S9: Supplementary Table on the Representativeness of Study Participants.

Category	
**Disease, problem, or condition under investigation**	HIV-associated cryptococcal meningitis (CM)
**Special considerations related to:**	
**Sex and gender**	HIV-associated CM affects men more than women (3:2 ratio)[1].
**Age**	The incidence of HIV-associated CM in sub-Saharan Africa is highest in the 35 to 40 year old age group, mirroring the peak age distributions of HIV-prevalence and prevalence of advanced HIV disease[1].
**Race or ethnic group**	CM occurs primarily in Black African individuals in sub-Saharan Africa[2].
**Geography**	HIV-associated CM occurs primarily in Low- and Middle-Income Countries (LMICs), with the majority of cases (approximately 73%) and deaths (approximately 75%) occurring in sub-Saharan Africa. The largest burden of disease is in southern and east Africa[2].
**Other considerations**	Ten-week mortality rates from HIV-associated cryptococcal meningitis in settings using amphotericin B based treatment in Africa have ranged from 30-45% in recent trials[3-5] and are up to 50% in routine care settings[6,7], with similar outcomes reported from LMIC settings in Asia and South America. Although the incidence of CM is lower in high income countries, several thousand cases still occur every year in the USA, where in-patient mortality rates are reported to be 10-15%[8].
**Overall representativeness of this trial**	The participants in the AMBITION-cm trial had the expected ratio of men to women, and an age distribution in keeping with that seen in the population developing CM across LMICs. All participants were Black Africans, with no participants recruited outside of Africa. The proportion of individuals who were antiretroviral therapy (ART) experienced (i.e. were currently taking or were previously taking ART) was higher than in previously reported cohorts of individuals with CM from across the African continent[9], but similar to the proportions reported in cohorts of individuals with advanced HIV disease in southern and East Africa during the study period[10]. A reduced Glasgow Coma Score (GCS) was seen in 28% of trial participants at presentation. This proportion is very similar to the proportion of individuals with low GCS seen in previous CM treatment trials performed from 2010 onwards in Africa and Asia, but lower than the 40-50% reported in routine care settings[9], possibly indicating than some of the sickest patients were unable to enrol in the trial due to dying before screening or randomization, being moribund, or were unable to consent.

**Methods and questions used to collect the information**	We have recently comprehensively reviewed the representativeness of participants in clinical trials for HIV-associated cryptococcal meningitis in a systematic review[9].

Table S9 References:

1. Tenforde MW, Mokomane M, Leeme T, et al. Advanced Human Immunodeficiency Virus Disease in Botswana Following Successful Antiretroviral Therapy Rollout: Incidence of and Temporal Trends in Cryptococcal Meningitis. Clin Infect Dis 2017;65:779-86.
2. Rajasingham R, Smith RM, Park BJ, et al. Global burden of disease of HIV-associated cryptococcal meningitis: an updated analysis. Lancet Infect Dis 2017.
3. Day JN, Chau TTH, Wolbers M, et al. Combination antifungal therapy for cryptococcal meningitis. N Engl J Med 2013;368:1291-302.
4. Beardsley J, Wolbers M, Kibengo FM, et al. Adjunctive Dexamethasone in HIV-Associated Cryptococcal Meningitis. N Engl J Med 2016;374:542-54.
5. Molloy SF, Kanyama C, Heyderman RS, et al. Antifungal Combinations for Treatment of Cryptococcal Meningitis in Africa. N Engl J Med 2018;378:1004-17.
6. Patel RKK, Leeme T, Azzo C, et al. High Mortality in HIV-Associated Cryptococcal Meningitis Patients Treated With Amphotericin B-Based Therapy Under Routine Care Conditions in Africa. Open Forum Infect Dis 2018;5:ofy267.
7. Tenforde MW, Gertz AM, Lawrence DS, et al. Mortality from HIV-associated meningitis in sub-Saharan Africa: a systematic review and meta-analysis. J Int AIDS Soc 2020;23:e25416.
8. Pyrgos V, Seitz AE, Steiner CA, Prevots DR, Williamson PR. Epidemiology of cryptococcal meningitis in the US: 1997-2009. PLoS One 2013;8:e56269.
9. Lawrence DS, Leeme T, Mosepele M, Harrison TS, Seeley J, Jarvis JN. Equity in clinical trials for HIV-associated cryptococcal meningitis: A systematic review of global representation and inclusion of patients and researchers. PLoS Negl Trop Dis 2021;15:e0009376.
10. Lawrence DS, Tenforde MW, Milton T, et al. The epidemiology of advanced HIV disease before and after universal art in Botswana. 28th Conference on Retroviruses and Opportunistic Infections (CROI) 2021;March 6-10, 2021, Virtual.

Table S10: Antiretroviral therapy management SOP

Management of antiretroviral therapy (ART) for participants in the AMBITION-*cm* trial

**ART naïve participant**	**Do not start ART** at admission	**Wait** for 4 – 6 weeks	**1st line ART regimen** as per local guidelines
**ART experienced participant** reported <u>not adherent</u> or <u>uncertain adherence</u>	**Stop ART** at admission	**Wait** for 4 – 6 weeks	**Re-start 1st, 2nd or 3rd line ART regimen*** After 4 weeks
**ART experienced participant** Reportedly <u>adherent</u> on ART > 6 months **	**Stop ART** at admission	**Wait** for 4 – 6 weeks	**Re-start 1st, 2nd or 3rd line ART regimen*** After 4 weeks
**ART experienced** Reportedly <u>adherent</u> on ART ≤ 6 months Recent introduction **or** re-introduction	**Continue ART** at admission	Consider unmasking IRIS diagnosis	**Continue 1st, 2nd or 3rd line ART regimen*** Check for CD4 count and HIV viral load

* Decision on ART regimen to re-start should be made according to local guidelines, HIV viral load, genotypic resistance testing if possible, patient's history. If it is considered likely that the patient has developed resistance to 1st line (e.g. NNRTI resistance), then restart with 2nd line containing boosted PI or DTG if possible.

** Unless documented to have a suppressed viral load at time of admission of within the month prior to admission, in which case continue ART

v1.2 25.10.2019
